# Di-*tert*-butyl *N*,*N*′-(octa­hydro­penta­lene-2,5-di­yl)dicarbamate

**DOI:** 10.1107/S1600536808014876

**Published:** 2008-05-24

**Authors:** Amol M. Kendhale, Rajesh G. Gonnade, Gangadhar J. Sanjayan

**Affiliations:** a Division of Organic Chemistry, National Chemical Laboratory, Pashan Road, Pune 411 008, India; b Center for Materials Characterization, National Chemical Laboratory, Pashan Road, Pune 411 008, India

## Abstract

In the molecule of the title compound, C_18_H_32_N_2_O_4_, the central bicyclo­[3.3.0]octane (octa­hydro­penta­lene) has a rigid ring junction. Both rings of the bicyclo­[3.3.0]octane unit adopt an envelope conformation, and the flexible *tert*-butyl­carbamoyl side chains each have an extended conformation. Such a constrained bicyclo­[3.3.0]octane aliphatic template is of inter­est with respect to the design of novel self-assembling motifs. Mol­ecules related by *c*-glide symmetry are linked *via* inter­molecular N—H⋯O hydrogen bonds, forming a two-dimensional layer structure. Neighboring layers are weakly associated along the *a* axis due to the close approach of the *tert*-butyl­carbamoyl groups (2.55 Å).

## Related literature

For related literature, see: Bertz *et al.* (1982 ▶); Kendhale *et al.* (2008 ▶); Yates *et al.* (1960 ▶); Yeo *et al.* (2006 ▶).
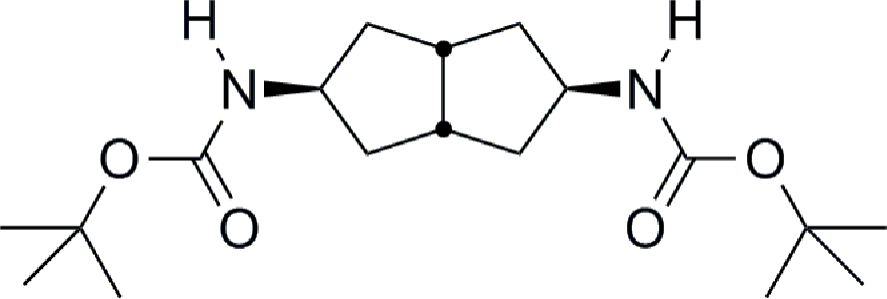


## Experimental

### 

#### Crystal data

C_18_H_32_N_2_O_4_
*M* *_r_* = 340.46
Monoclinic, 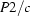

*a* = 33.161 (17) Å
*b* = 6.060 (3) Å
*c* = 9.926 (5) Å
β = 95.594 (9)°
*V* = 1985.2 (18) Å^3^
*Z* = 4
Mo *K*α radiation
μ = 0.08 mm^−1^
*T* = 297 (2) K
0.64 × 0.13 × 0.08 mm

#### Data collection

Bruker SMART APEX diffractometer
Absorption correction: multi-scan (*SADABS*; Bruker, 2003 ▶) *T*_min_ = 0.951, *T*_max_ = 0.994
9395 measured reflections
3479 independent reflections
2863 reflections with *I* > 2σ(*I*)
*R*_int_ = 0.026

#### Refinement

*R*[*F* ^2^ > 2σ(*F* ^2^)] = 0.056
*wR*(*F* ^2^) = 0.133
*S* = 1.08
3479 reflections
271 parameters
H atoms treated by a mixture of independent and constrained refinement
Δρ_max_ = 0.17 e Å^−3^
Δρ_min_ = −0.14 e Å^−3^

### 

Data collection: *SMART* (Bruker, 2003 ▶); cell refinement: *SAINT* (Bruker, 2003 ▶); data reduction: *SAINT*; program(s) used to solve structure: *SHELXS97* (Sheldrick, 2008 ▶); program(s) used to refine structure: *SHELXL97* (Sheldrick, 2008 ▶); molecular graphics: *ORTEP-3* (Farrugia, 1997 ▶); software used to prepare material for publication: *SHELXTL* (Sheldrick, 2008 ▶).

## Supplementary Material

Crystal structure: contains datablocks I, global. DOI: 10.1107/S1600536808014876/wk2086sup1.cif

Structure factors: contains datablocks I. DOI: 10.1107/S1600536808014876/wk2086Isup2.hkl

Additional supplementary materials:  crystallographic information; 3D view; checkCIF report

## Figures and Tables

**Table 1:** Hydrogen-bond geometry (Å, °)

*D*—H⋯*A*	*D*—H	H⋯*A*	*D*⋯*A*	*D*—H⋯*A*
N2—H2⋯O3^i^	0.86	2.11	2.954 (3)	167
N1—H1⋯O1^ii^	0.86	2.19	3.022 (3)	162

Symmetry codes: (i) 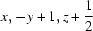; (ii) 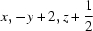.

**Fig. 1.**
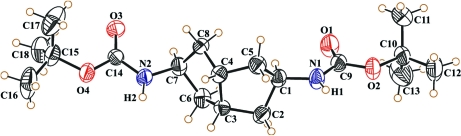
Molecular structure of (I), with atom labels and 50% probability displacement ellipsoids for non-H atoms.

**Fig. 2.**
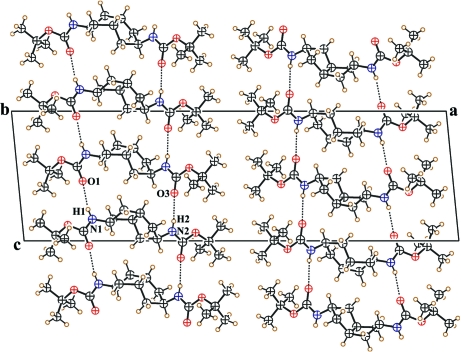
Molecular packing viewed down the b axis, showing the layered arrangement of the molecules linked via N—H···O hydrogen bonds.

## supplementary crystallographic information

### Comment

The skeleton of bicyclo[3.3.0]octane is interesting because it has a rigid ring junction as well as conformationally flexible side groups (Bertz *et al.*, 1982; Yates *et al.*, 1960). Depending on the substituents, it can adopt one of three different conformations in a given circumstance (Yeo *et al.*, 2006). In the context of our interest in extending the applicability of bicyclo[3.3.0]octane as a self-assembling motif (Kendhale *et al.*, 2008), the title compound (I) has been synthesized and here we report its crystal structure.

The two five-membered rings of the bicyclo[3.3.0]octane subunit adopt an *exo/endo* envelope conformation, while the flexible *tert*-Butylcarbamoyl group takes an extended conformation (Fig. 1).

In the crystal, molecules related by c-glide symmetry are linked *via* intermolecular N—H···O hydrogen bonds (Table 1) forming a layered arrangement  (Fig.2). These layers are weakly associated along the *a* axis due to the close approach of the bulkier *tert-*butylcarbamoy group (2.55 Å).

### Experimental

Tetramethylbicyclo[3.3.0]octane-3,7-dione-2,4,6,8-tetracarboxylate, bicyclo[3.3.0]octane-3,7-dione and 2, 5-dihydroxy-bicyclo[3.3.0]octane were prepared according to the literature procedure (Bertz *et al.*, 1982; Yeo *et al.*, 2006). The 2, 5-dihydroxy-bicyclo[3.3.0]octane (3.86 g, 27.183 mmol) was treated with methanesulfonyl chloride (6.31 ml, 9.34 g, 81.549 mmol) and triethyl amine (11.36 ml, 8.25 g, 81.549 mmol) in DCM (50 ml) at room temperature for 12 h to obtain 2,5-dimethanesulfonyloxy bicyclo[3.3.0]octane. Nucleophilic displacement of 2,5-dimethanesulfonyloxy bicyclo[3.3.0]octane (6 g, 20.134 mmol) by sodium azide (13.08 g, 201.34 mmol) in DMF (40 ml) at 343 K for 24 h, delivered 2,5-diazido-bicyclo[3.3.0]octane. The 2,5-diazido-bicyclo[3.3.0]octane (0.5 g, 2.6041 mmol) was hydrogenated in the presence of Pd/c-methanol (20 ml) system, and *in situ* protection with *tert*-Butyl Dicarbonate (Boc)2O, (1.7 g, 7.812 mmol), afforded the required 5-*tert*-Butoxycarbonylamino-octahydro-pentalen-2-yl)-carbamic acid *tert*-butyl ester (0.61 g, 69%). Colourless needles suitable for X-ray diffraction were obtained by slow evaporation of a solution in methanol-ethyl acetate (1:4) mixture at room temperature.

### Refinement

The H atoms bonded to bicyclo[3.3.0]octane unit were located in a difference Fourier map and refined isotropically. Other H atoms bonded to N atoms and *tert*-butyl group were placed in geometrically idealized positions with N—H = 0.86 Å (for NH) and C—H = 0.96 Å (for methyl H) and constrained to ride on their parent atoms with *U*iso(H) = *xU*_eq_(C,*N*), where *x* = 1.2 for NH amd *x* = 1.5 for methyl H.

### Crystal data

**Table:**

C_18_H_32_N_2_O_4_	*F*_000_ = 744
*M**_r_* = 340.46	*D*_x_ = 1.139 Mg m^−^^3^
Monoclinic, *P*2/*c*	Mo *K*α radiation λ = 0.71073 Å
Hall symbol: -P 2yc	Cell parameters from 3113 reflections
*a* = 33.161 (17) Å	θ = 2.5–25.4º
*b* = 6.060 (3) Å	µ = 0.08 mm^−^^1^
*c* = 9.926 (5) Å	*T* = 297 (2) K
β = 95.594 (9)º	Needle, colourless
*V* = 1985.2 (18) Å^3^	0.64 × 0.13 × 0.08 mm
*Z* = 4	

### Data collection

**Table:**

Bruker SMART APEX diffractometer	3479 independent reflections
Radiation source: fine-focus sealed tube	2863 reflections with *I* > 2σ(*I*)
Monochromator: graphite	*R*_int_ = 0.026
*T* = 297(2) K	θ_max_ = 25.0º
φ and ω scans	θ_min_ = 1.2º
Absorption correction: multi-scan(SADABS; Bruker, 2003)	*h* = −39→29
*T*_min_ = 0.951, *T*_max_ = 0.994	*k* = −7→7
9395 measured reflections	*l* = −10→11

### Refinement

**Table:**

Refinement on *F*^2^	Secondary atom site location: difference Fourier map
Least-squares matrix: full	Hydrogen site location: geom, difmap for bicyclo unit
*R*[*F*^2^ > 2σ(*F*^2^)] = 0.056	H atoms treated by a mixture of independent and constrained refinement
*wR*(*F*^2^) = 0.133	*w* = 1/[σ^2^(*F*_o_^2^) + (0.0452*P*)^2^ + 0.984*P*] where *P* = (*F*_o_^2^ + 2*F*_c_^2^)/3
*S* = 1.08	(Δ/σ)_max_ < 0.001
3479 reflections	Δρ_max_ = 0.17 e Å^−^^3^
271 parameters	Δρ_min_ = −0.14 e Å^−^^3^
Primary atom site location: structure-invariant direct methods	Extinction correction: none

### Special details

**Table:**

Geometry. All e.s.d.'s (except the e.s.d. in the dihedral angle between two l.s. planes) are estimated using the full covariance matrix. The cell e.s.d.'s are taken into account individually in the estimation of e.s.d.'s in distances, angles and torsion angles; correlations between e.s.d.'s in cell parameters are only used when they are defined by crystal symmetry. An approximate (isotropic) treatment of cell e.s.d.'s is used for estimating e.s.d.'s involving l.s. planes.
Refinement. Refinement of *F*^2^ against ALL reflections. The weighted *R*-factor *wR* and goodness of fit *S* are based on *F*^2^, conventional *R*-factors *R* are based on *F*, with *F* set to zero for negative *F*^2^. The threshold expression of *F*^2^ > σ(*F*^2^) is used only for calculating *R*-factors(gt) *etc*. and is not relevant to the choice of reflections for refinement. *R*-factors based on *F*^2^ are statistically about twice as large as those based on *F*, and *R*- factors based on ALL data will be even larger.

### Fractional atomic coordinates and isotropic or equivalent isotropic displacement parameters (Å^2^)

**Table:**

	*x*	*y*	*z*	*U*_iso_*/*U*_eq_	
O1	0.14804 (5)	1.1114 (3)	0.46136 (15)	0.0640 (5)	
O2	0.10387 (4)	1.1306 (3)	0.62262 (15)	0.0567 (4)	
O3	0.35645 (5)	0.4521 (3)	0.36953 (14)	0.0614 (5)	
O4	0.39622 (4)	0.3724 (3)	0.56279 (14)	0.0559 (4)	
N1	0.16176 (5)	0.9541 (3)	0.66747 (17)	0.0496 (5)	
H1	0.1525	0.9274	0.7438	0.060*	
N2	0.34243 (5)	0.5850 (3)	0.57189 (16)	0.0460 (5)	
H2	0.3496	0.5892	0.6575	0.055*	
C1	0.20180 (6)	0.8722 (4)	0.6444 (2)	0.0421 (5)	
C2	0.23645 (7)	0.9724 (4)	0.7371 (3)	0.0526 (6)	
C3	0.27303 (6)	0.8282 (3)	0.7141 (2)	0.0395 (5)	
C4	0.25453 (6)	0.6036 (3)	0.6631 (2)	0.0396 (5)	
C5	0.20867 (7)	0.6266 (4)	0.6674 (3)	0.0485 (5)	
C6	0.29869 (7)	0.9084 (4)	0.6030 (2)	0.0471 (5)	
C7	0.30653 (6)	0.7070 (4)	0.5165 (2)	0.0439 (5)	
C8	0.26760 (7)	0.5747 (4)	0.5201 (2)	0.0437 (5)	
C9	0.13895 (6)	1.0694 (4)	0.5737 (2)	0.0450 (5)	
C10	0.07445 (7)	1.2714 (4)	0.5428 (2)	0.0522 (6)	
C11	0.05791 (8)	1.1566 (5)	0.4146 (3)	0.0763 (8)	
H11A	0.0788	1.1449	0.3545	0.114*	
H11B	0.0356	1.2399	0.3716	0.114*	
H11C	0.0487	1.0116	0.4360	0.114*	
C12	0.04103 (8)	1.2937 (6)	0.6378 (3)	0.0875 (10)	
H12A	0.0302	1.1505	0.6547	0.131*	
H12B	0.0198	1.3864	0.5965	0.131*	
H12C	0.0521	1.3582	0.7218	0.131*	
C13	0.09321 (10)	1.4901 (5)	0.5166 (4)	0.0927 (10)	
H13A	0.1068	1.5463	0.5994	0.139*	
H13B	0.0725	1.5917	0.4825	0.139*	
H13C	0.1124	1.4724	0.4511	0.139*	
C14	0.36414 (6)	0.4672 (4)	0.4910 (2)	0.0415 (5)	
C15	0.42463 (7)	0.2332 (4)	0.4955 (2)	0.0554 (6)	
C16	0.45595 (10)	0.1763 (7)	0.6134 (3)	0.1111 (14)	
H16A	0.4667	0.3098	0.6547	0.167*	
H16B	0.4775	0.0925	0.5806	0.167*	
H16C	0.4433	0.0908	0.6791	0.167*	
C17	0.40278 (10)	0.0335 (5)	0.4356 (4)	0.0995 (12)	
H17A	0.3882	−0.0360	0.5030	0.149*	
H17B	0.4221	−0.0686	0.4051	0.149*	
H17C	0.3841	0.0775	0.3604	0.149*	
C18	0.44483 (8)	0.3633 (5)	0.3914 (3)	0.0732 (8)	
H18A	0.4255	0.3942	0.3153	0.110*	
H18B	0.4668	0.2790	0.3618	0.110*	
H18C	0.4550	0.4994	0.4306	0.110*	
H3	0.2897 (6)	0.808 (3)	0.798 (2)	0.043 (6)*	
H4	0.2652 (6)	0.486 (4)	0.720 (2)	0.050 (6)*	
H7	0.3117 (6)	0.747 (3)	0.422 (2)	0.047 (6)*	
H1A	0.2045 (6)	0.908 (3)	0.555 (2)	0.045 (6)*	
H2A	0.2412 (8)	1.128 (5)	0.717 (3)	0.074 (8)*	
H5A	0.1936 (8)	0.540 (5)	0.598 (3)	0.080 (9)*	
H6A	0.3249 (8)	0.982 (4)	0.643 (3)	0.070 (7)*	
H8A	0.2709 (6)	0.416 (4)	0.495 (2)	0.044 (6)*	
H2B	0.2287 (8)	0.959 (4)	0.836 (3)	0.073 (8)*	
H5B	0.2002 (7)	0.592 (4)	0.758 (2)	0.052 (6)*	
H6B	0.2824 (7)	1.009 (4)	0.544 (2)	0.058 (7)*	
H8B	0.2473 (6)	0.636 (3)	0.456 (2)	0.038 (5)*	

### Atomic displacement parameters (Å^2^)

**Table:**

	*U*^11^	*U*^22^	*U*^33^	*U*^12^	*U*^13^	*U*^23^
O1	0.0590 (10)	0.0951 (14)	0.0394 (9)	0.0194 (9)	0.0125 (7)	0.0133 (9)
O2	0.0453 (9)	0.0790 (12)	0.0467 (9)	0.0222 (8)	0.0095 (7)	0.0101 (8)
O3	0.0549 (10)	0.0956 (13)	0.0333 (9)	0.0147 (9)	0.0019 (7)	−0.0137 (8)
O4	0.0495 (9)	0.0773 (11)	0.0413 (8)	0.0235 (8)	0.0055 (7)	−0.0050 (8)
N1	0.0437 (10)	0.0693 (13)	0.0377 (9)	0.0157 (9)	0.0131 (8)	0.0096 (9)
N2	0.0442 (10)	0.0644 (12)	0.0296 (8)	0.0127 (9)	0.0051 (7)	−0.0014 (8)
C1	0.0410 (11)	0.0513 (13)	0.0353 (11)	0.0063 (10)	0.0095 (9)	0.0038 (10)
C2	0.0498 (13)	0.0465 (14)	0.0628 (15)	−0.0001 (11)	0.0122 (11)	−0.0151 (12)
C3	0.0394 (11)	0.0432 (12)	0.0356 (11)	−0.0004 (9)	0.0022 (9)	−0.0018 (9)
C4	0.0439 (12)	0.0335 (11)	0.0415 (11)	0.0030 (9)	0.0049 (9)	0.0040 (9)
C5	0.0428 (12)	0.0478 (13)	0.0559 (14)	−0.0051 (10)	0.0092 (11)	−0.0022 (11)
C6	0.0429 (12)	0.0424 (12)	0.0569 (14)	0.0003 (10)	0.0093 (10)	0.0061 (11)
C7	0.0412 (11)	0.0570 (14)	0.0341 (11)	0.0087 (10)	0.0072 (9)	0.0072 (10)
C8	0.0448 (12)	0.0450 (13)	0.0406 (12)	0.0084 (10)	0.0001 (9)	−0.0078 (10)
C9	0.0416 (12)	0.0544 (13)	0.0395 (12)	0.0069 (10)	0.0058 (9)	−0.0011 (10)
C10	0.0436 (12)	0.0594 (14)	0.0523 (13)	0.0136 (11)	−0.0024 (10)	0.0019 (11)
C11	0.0626 (16)	0.089 (2)	0.0735 (18)	0.0099 (15)	−0.0139 (14)	−0.0117 (16)
C12	0.0612 (17)	0.122 (3)	0.0797 (19)	0.0425 (18)	0.0100 (14)	0.0030 (19)
C13	0.079 (2)	0.0664 (19)	0.128 (3)	0.0028 (16)	−0.0131 (19)	0.0090 (19)
C14	0.0361 (11)	0.0540 (13)	0.0348 (11)	0.0010 (10)	0.0044 (8)	−0.0033 (9)
C15	0.0534 (14)	0.0604 (15)	0.0551 (13)	0.0157 (12)	0.0195 (11)	0.0013 (12)
C16	0.096 (2)	0.159 (4)	0.081 (2)	0.084 (2)	0.0219 (18)	0.025 (2)
C17	0.104 (2)	0.0595 (18)	0.145 (3)	−0.0050 (17)	0.063 (2)	−0.018 (2)
C18	0.0549 (15)	0.0820 (19)	0.0867 (19)	0.0037 (14)	0.0270 (14)	0.0087 (16)

### Geometric parameters (Å, °)

**Table:**

O1—C9	1.210 (3)	C7—C8	1.523 (3)
O2—C9	1.355 (2)	C7—H7	1.00 (2)
O2—C10	1.469 (3)	C8—H8A	1.00 (2)
O3—C14	1.211 (2)	C8—H8B	0.95 (2)
O4—C14	1.350 (2)	C10—C13	1.497 (4)
O4—C15	1.472 (3)	C10—C11	1.506 (3)
N1—C9	1.337 (3)	C10—C12	1.530 (3)
N1—C1	1.456 (3)	C11—H11A	0.9600
N1—H1	0.8600	C11—H11B	0.9600
N2—C14	1.336 (3)	C11—H11C	0.9600
N2—C7	1.462 (3)	C12—H12A	0.9600
N2—H2	0.8600	C12—H12B	0.9600
C1—C5	1.520 (3)	C12—H12C	0.9600
C1—C2	1.526 (3)	C13—H13A	0.9600
C1—H1A	0.92 (2)	C13—H13B	0.9600
C2—C3	1.530 (3)	C13—H13C	0.9600
C2—H2A	0.98 (3)	C15—C17	1.503 (4)
C2—H2B	1.04 (3)	C15—C18	1.508 (3)
C3—C6	1.536 (3)	C15—C16	1.527 (4)
C3—C4	1.557 (3)	C16—H16A	0.9600
C3—H3	0.96 (2)	C16—H16B	0.9600
C4—C5	1.532 (3)	C16—H16C	0.9600
C4—C8	1.534 (3)	C17—H17A	0.9600
C4—H4	0.96 (2)	C17—H17B	0.9600
C5—H5A	0.96 (3)	C17—H17C	0.9600
C5—H5B	0.99 (2)	C18—H18A	0.9600
C6—C7	1.529 (3)	C18—H18B	0.9600
C6—H6A	1.02 (3)	C18—H18C	0.9600
C6—H6B	0.97 (2)		
			
C9—O2—C10	120.93 (17)	H8A—C8—H8B	107.1 (17)
C14—O4—C15	120.70 (16)	O1—C9—N1	125.17 (19)
C9—N1—C1	122.09 (17)	O1—C9—O2	124.89 (19)
C9—N1—H1	119.0	N1—C9—O2	109.93 (18)
C1—N1—H1	119.0	O2—C10—C13	110.0 (2)
C14—N2—C7	120.73 (17)	O2—C10—C11	110.8 (2)
C14—N2—H2	119.6	C13—C10—C11	112.7 (2)
C7—N2—H2	119.6	O2—C10—C12	101.63 (18)
N1—C1—C5	115.83 (18)	C13—C10—C12	111.5 (2)
N1—C1—C2	114.47 (18)	C11—C10—C12	109.6 (2)
C5—C1—C2	101.90 (19)	C10—C11—H11A	109.5
N1—C1—H1A	104.2 (13)	C10—C11—H11B	109.5
C5—C1—H1A	110.1 (13)	H11A—C11—H11B	109.5
C2—C1—H1A	110.4 (13)	C10—C11—H11C	109.5
C1—C2—C3	104.11 (18)	H11A—C11—H11C	109.5
C1—C2—H2A	112.7 (16)	H11B—C11—H11C	109.5
C3—C2—H2A	111.8 (16)	C10—C12—H12A	109.5
C1—C2—H2B	107.3 (14)	C10—C12—H12B	109.5
C3—C2—H2B	111.8 (14)	H12A—C12—H12B	109.5
H2A—C2—H2B	109 (2)	C10—C12—H12C	109.5
C2—C3—C6	115.44 (19)	H12A—C12—H12C	109.5
C2—C3—C4	104.78 (17)	H12B—C12—H12C	109.5
C6—C3—C4	105.77 (17)	C10—C13—H13A	109.5
C2—C3—H3	110.0 (12)	C10—C13—H13B	109.5
C6—C3—H3	110.2 (12)	H13A—C13—H13B	109.5
C4—C3—H3	110.3 (13)	C10—C13—H13C	109.5
C5—C4—C8	113.99 (19)	H13A—C13—H13C	109.5
C5—C4—C3	105.79 (17)	H13B—C13—H13C	109.5
C8—C4—C3	105.21 (17)	O3—C14—N2	124.39 (19)
C5—C4—H4	111.3 (13)	O3—C14—O4	124.89 (19)
C8—C4—H4	109.9 (13)	N2—C14—O4	110.70 (17)
C3—C4—H4	110.4 (13)	O4—C15—C17	109.6 (2)
C1—C5—C4	102.69 (17)	O4—C15—C18	111.0 (2)
C1—C5—H5A	111.6 (17)	C17—C15—C18	112.3 (2)
C4—C5—H5A	112.2 (16)	O4—C15—C16	101.48 (19)
C1—C5—H5B	106.9 (13)	C17—C15—C16	112.8 (3)
C4—C5—H5B	112.0 (13)	C18—C15—C16	109.2 (2)
H5A—C5—H5B	111 (2)	C15—C16—H16A	109.5
C7—C6—C3	106.68 (18)	C15—C16—H16B	109.5
C7—C6—H6A	112.4 (14)	H16A—C16—H16B	109.5
C3—C6—H6A	111.9 (14)	C15—C16—H16C	109.5
C7—C6—H6B	105.8 (14)	H16A—C16—H16C	109.5
C3—C6—H6B	108.6 (14)	H16B—C16—H16C	109.5
H6A—C6—H6B	111 (2)	C15—C17—H17A	109.5
N2—C7—C8	112.69 (19)	C15—C17—H17B	109.5
N2—C7—C6	111.67 (18)	H17A—C17—H17B	109.5
C8—C7—C6	102.47 (17)	C15—C17—H17C	109.5
N2—C7—H7	105.5 (12)	H17A—C17—H17C	109.5
C8—C7—H7	111.8 (12)	H17B—C17—H17C	109.5
C6—C7—H7	112.9 (12)	C15—C18—H18A	109.5
C7—C8—C4	106.16 (17)	C15—C18—H18B	109.5
C7—C8—H8A	112.7 (12)	H18A—C18—H18B	109.5
C4—C8—H8A	112.7 (12)	C15—C18—H18C	109.5
C7—C8—H8B	109.0 (12)	H18A—C18—H18C	109.5
C4—C8—H8B	109.2 (12)	H18B—C18—H18C	109.5
			
C9—N1—C1—C5	−126.3 (2)	C3—C6—C7—C8	34.0 (2)
C9—N1—C1—C2	115.6 (2)	N2—C7—C8—C4	83.2 (2)
N1—C1—C2—C3	168.58 (18)	C6—C7—C8—C4	−36.9 (2)
C5—C1—C2—C3	42.8 (2)	C5—C4—C8—C7	141.38 (19)
C1—C2—C3—C6	91.7 (2)	C3—C4—C8—C7	25.9 (2)
C1—C2—C3—C4	−24.2 (2)	C1—N1—C9—O1	2.5 (4)
C2—C3—C4—C5	−3.1 (2)	C1—N1—C9—O2	−178.18 (19)
C6—C3—C4—C5	−125.53 (19)	C10—O2—C9—O1	−4.6 (4)
C2—C3—C4—C8	117.87 (19)	C10—O2—C9—N1	176.08 (19)
C6—C3—C4—C8	−4.6 (2)	C9—O2—C10—C13	−61.7 (3)
N1—C1—C5—C4	−169.18 (18)	C9—O2—C10—C11	63.6 (3)
C2—C1—C5—C4	−44.3 (2)	C9—O2—C10—C12	180.0 (2)
C8—C4—C5—C1	−85.8 (2)	C7—N2—C14—O3	0.3 (3)
C3—C4—C5—C1	29.3 (2)	C7—N2—C14—O4	178.76 (18)
C2—C3—C6—C7	−133.66 (19)	C15—O4—C14—O3	−1.7 (3)
C4—C3—C6—C7	−18.3 (2)	C15—O4—C14—N2	179.80 (19)
C14—N2—C7—C8	93.1 (2)	C14—O4—C15—C17	−63.5 (3)
C14—N2—C7—C6	−152.15 (19)	C14—O4—C15—C18	61.1 (3)
C3—C6—C7—N2	−86.9 (2)	C14—O4—C15—C16	177.1 (2)

### Hydrogen-bond geometry (Å, °)

**Table:**

*D*—H···*A*	*D*—H	H···*A*	*D*···*A*	*D*—H···*A*
N2—H2···O3^i^	0.86	2.11	2.954 (3)	167
N1—H1···O1^ii^	0.86	2.19	3.022 (3)	162

Symmetry codes: (i) *x*, −*y*+1, *z*+1/2; (ii) *x*, −*y*+2, *z*+1/2.
